# As a mature scientific discipline animal welfare must be subject to debate and opinion

**DOI:** 10.1017/awf.2023.89

**Published:** 2023-10-12

**Authors:** Huw D. R. Golledge, Birte L. Nielsen

**Affiliations:** Universities Federation for Animal Welfare, Wheathampstead, United Kingdom

**Keywords:** animal welfare, animal welfare science, debate, Five Domains, opinion, stunning

Our tagline at the Universities Federation for Animal Welfare (UFAW) is “Science in the Service of Animal Welfare”. With this, we argue that the best way to improve animal welfare is to use science to discover what animals need and want, develop ways to provide for those needs and wants and then disseminate and implement the findings. Over recent decades, animal welfare has gradually established itself as a mature scientific discipline and we see UFAW’s scientific journal *Animal Welfare* as a key route for the dissemination of high-quality, scientifically rigorous animal welfare research. However, science is rarely as clear-cut as some may imagine and we therefore want to establish *Animal Welfare* as a forum for evidence-based debate too, something which is essential to scientific progress. By challenging scientific claims, the quality of the evidence is tested allowing more robust conclusions to be formed. To this end, *Animal Welfare* has recently published two Opinion Papers (Zivotofsky 2023, Hampton et al. 2023), some of the first to appear in this Journal.

Both articles are likely, albeit in very different ways, to be controversial, but we consider them an important part of the scientific discourse. Publishing them in *Animal Welfare* is one way that the Journal can achieve its fundamental purpose which is to ensure that animal welfare research can lead to real world improvements in the lives of animals. In both cases, the Opinion Papers have been subject to the same rigorous peer review that we apply to primary research, ensuring that the debate remains firmly grounded in scientific evidence.

In the first paper, Zivotofsky (2023) outlines a critique of a novel stunning method for livestock which has been developed over recent years. Diathermic Syncope® (DTS) is a microwave-based method designed to render livestock animals unconscious prior to slaughter. The author takes issue with both the name of the technique, which he argues does not accurately represent the mechanism by which unconsciousness is achieved, as well as claims for its animal welfare benefits. Since these purported benefits (and the name itself) can be used to advocate for the technique’s acceptability to those Muslim and Jewish religious authorities and consumers who do not accept other methods of pre-slaughter stunning, the paper is likely to be contentious. However, the arguments in the paper are well constructed and supported by scientific evidence.

In the second paper, Hampton and colleagues (Hampton et al. 2023) consider whether an increasingly popular method for assessing the welfare impacts of interventions which impact upon animals has the kind of reliability and scientific rigour one would expect. The Five Domains method has been employed as a simple and rapid way of assessing consensus (typically amongst those with expertise in a particular field) on the animal welfare impacts of various interventions. The aim of such studies is laudable – to determine the best way to treat animals – but if, as Hampton and colleagues argue, the method as currently applied is not as rigorous as intended, it is possible that the wrong conclusions could be reached. Studies using a Five Domains approach are used to form policy positions and to advocate for changes which could have real-world consequences for the target species (and, as the authors argue, also for non-target species which the studies may not even consider). Crucially, alongside their critique Hampton and colleagues also propose some potential improvements and alternative approaches which they argue could do a better job.

A common theme in both Opinion Papers is that they bring an early challenge to approaches which the authors suggest may not guarantee the animal welfare benefits claimed. We are happy to allow our readers to form their own opinions and we look forward to seeing the reactions to these Opinion Papers. In both cases, we are open to publishing equally well-argued counter opinions should those whose work is scrutinised or others wish to respond. As an open-access journal, we see *Animal Welfare* as an ideal forum for reasoned, evidence-based debate of key issues in animal welfare science and look forward to publishing more such critical Opinion Papers in future.
