# As a mature scientific discipline animal welfare must be subject to debate and opinion – ERRATUM

**DOI:** 10.1017/awf.2023.96

**Published:** 2023-11-08

**Authors:** Huw D. R. Golledge, Birte L. Nielsen

The publisher apologises that upon publication of this article the name Hemsworth, PH was incorrectly spelled as Hemosworth in the reference:

Hampton, JO, Hemsworth LM, Hemsworth, PH, Hyndman, TJ and Sandøe P 2023 Rethinking the utility of the Five Domains model. Animal Welfare, 32, e62, https://doi.org/10.1017/awf.2023.84

This has been corrected in the article
